# The upregulation of thiamine (vitamin B_1_) biosynthesis in *Arabidopsis thaliana *seedlings under salt and osmotic stress conditions is mediated by abscisic acid at the early stages of this stress response

**DOI:** 10.1186/1471-2229-12-2

**Published:** 2012-01-03

**Authors:** Maria Rapala-Kozik, Natalia Wolak, Marta Kujda, Agnieszka K Banas

**Affiliations:** 1 Department of Analytical Biochemistry, Faculty of Biochemistry, Biophysics and Biotechnology, Jagiellonian University, Gronostajowa 7, Kraków, Poland; 2 Department of Plant Biotechnology, Faculty of Biochemistry, Biophysics and Biotechnology, Jagiellonian University, Gronostajowa 7, Kraków, Poland

## Abstract

**Background:**

Recent reports suggest that vitamin B_1 _(thiamine) participates in the processes underlying plant adaptations to certain types of abiotic and biotic stress, mainly oxidative stress. Most of the genes coding for enzymes involved in thiamine biosynthesis in *Arabidopsis thaliana *have been identified. In our present study, we examined the expression of thiamine biosynthetic genes, of genes encoding thiamine diphosphate-dependent enzymes and the levels of thiamine compounds during the early (sensing) and late (adaptation) responses of Arabidopsis seedlings to oxidative, salinity and osmotic stress. The possible roles of plant hormones in the regulation of the thiamine contribution to stress responses were also explored.

**Results:**

The expression of Arabidopsis genes involved in the thiamine diphosphate biosynthesis pathway, including that of *THI1*, *THIC*, *TH1 *and *TPK*, was analyzed for 48 h in seedlings subjected to NaCl or sorbitol treatment. These genes were found to be predominantly up-regulated in the early phase (2-6 h) of the stress response. The changes in these gene transcript levels were further found to correlate with increases in thiamine and its diphosphate ester content in seedlings, as well as with the enhancement of gene expression for enzymes which require thiamine diphosphate as a cofactor, mainly α-ketoglutarate dehydrogenase, pyruvate dehydrogenase and transketolase. In the case of the phytohormones including the salicylic, jasmonic and abscisic acids which are known to be involved in plant stress responses, only abscisic acid was found to significantly influence the expression of thiamine biosynthetic genes, the thiamine diphosphate levels, as well as the expression of genes coding for main thiamine diphosphate-dependent enzymes. Using Arabidopsis mutant plants defective in abscisic acid production, we demonstrate that this phytohormone is important in the regulation of *THI1 *and *THIC *gene expression during salt stress but that the regulatory mechanisms underlying the osmotic stress response are more complex.

**Conclusions:**

On the basis of the obtained results and earlier reported data, a general model is proposed for the involvement of the biosynthesis of thiamine compounds and thiamine diphosphate-dependent enzymes in abiotic stress sensing and adaptation processes in plants. A possible regulatory role of abscisic acid in the stress sensing phase is also suggested by these data.

## Background

**Figure 1:**
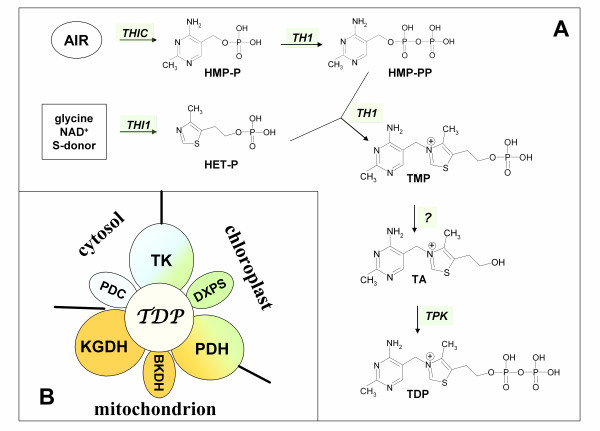
**Schematic representation of thiamine biosynthesis in plants (**A**) and subcellular localization of the major thiamine diphosphate-incorporating enzymes (**B**)**. The Arabidopsis genes encoding the enzymes which catalyze the biosynthetic reactions shown are specified. AIR, 5-aminoimidazole ribonucleotide; HET-P, 4-methyl-5-(2-hydroxyethyl)thiazole phosphate; HMP-P/HMP-PP, 4-amino-2-methyl-5-hydroxymethylpyrimidine monophosphate/diphosphate; TA, thiamine; TMP, thiamine monophosphate; TDP, thiamine diphosphate; PDC, pyruvate decarboxylase; PDH, pyruvate dehydrogenase; KGDH, α-ketoglutarate dehydrogenase; BKDH, branched-chain α-ketoacid dehydrogenase; TK, transketolase; DXPS, 1-deoxy-D-xylulose-5-phosphate synthase.

Vitamin B_1 _(thiamine) is essential for the proper functioning of all living organisms as it plays important roles in carbohydrate catabolism, NADPH and ATP synthesis and in the formation of nucleic acids. The biologically active form of thiamine is its diphosphate analog (TDP) which functions as a cofactor for a number of critical enzymes (Figure 1A) including pyruvate decarboxylase (PDC), pyruvate dehydrogenase (PDH), α-ketoglutarate dehydrogenase (KGDH), branched-chain α-ketoacid dehydrogenase (BKDH), transketolase (TK), acetolactate synthase (AHAS) and 1-deoxy-D-xylulose-5-phosphate synthase (DXPS) [1]. Plants can synthesize TDP *de novo *from simple precursors via biosynthetic pathways that bear the hallmarks of the systems utilized by both bacteria and yeast [2-5]. In this regard, the early stages of TDP biosynthesis include two parallel pathways. One is similar to the mechanism found in bacteria and leads from 5-aminoimidazole ribonucleotide (AIR) to the pyrimidine moiety of thiamine (4-amino-2-methyl-5-hydroxymethylpyrimidine monophosphate, HMP-P) and engages only a single enzyme, a product of the *THIC *gene that has been characterized in *Arabidopsis thaliana *[6]. In the second of the parallel pathways, the thiazole moiety of thiamine (4-methyl-5-(2-hydroxyethyl)-thiazole phosphate, HET-P) is synthesized from glycine, NAD^+ ^and a sulfur donor protein, in a similar manner to the system utilized in yeast [7]. The major enzyme involved in the HET-P formation is encoded by the *THI1 *gene, which has now been characterized in both Arabidopsis and *Zea mays *[8,9].

Two further thiamine biosynthetic steps include an additional phosphorylation of HMP-P to 4-amino-2-methyl-5-hydroxymethylpyrimidine diphosphate (HMP-PP) and the condensation of HMP-PP with HET-P to form thiamine monophosphate (TMP). These two reactions are catalyzed by a single protein, the product of the *TH1 *gene in Arabidopsis [10] or *THI3 *gene in *Z. mays *[11], which is thus a bifunctional enzyme with both HMP-P kinase and TMP synthase activities. TMP does not possess any recognized physiological function to date but constitutes a thiamine resource or transitional stage for further TDP synthesis. In the latter case, an unphosphorylated form of thiamine is required. As has been shown previously in *Z. mays *[12], TMP is dephosphorylated by a relatively unspecific phosphatase which can also use TDP as a substrate. All of the steps described above proceed in chloroplasts [13], but the process of thiamine conversion to TDP occurs in the cytosol and is catalyzed by thiamine pyrophosphokinase, encoded by the *TPK *genes [14]. To fulfill its biological function, TDP must be transported to chloroplasts or mitochondria but the required transporters have not yet been identified. The thiamine biosynthesis pathway in plants is precisely regulated through TDP-dependent riboswitch mechanisms on the *THIC *gene and in ancient plant taxa also the *THI1 *gene levels [15].

It has been well documented that thiamine biosynthesis is activated during plant adaptation responses to persistent abiotic stress conditions such as salting and flooding [16], cold, heat and drought [17,18], and oxidative stress [19,20]. However, it has not yet been determined how thiamine biosynthesis and activation responds to the above stimuli at the early stages of the corresponding stress responses (sensing). In our current study, we further characterize the role of thiamine biosynthesis upregulation in the early response to oxidative, osmotic and salt stress in plants. In the experiments, we analyze changes in the expression levels of all thiamine-biosynthetic genes and of genes encoding the major TDP-dependent enzymes such as TK, KGDH, PDH, PDC and DXPS in Arabidopsis seedlings. We also show for the first time that abscisic acid (ABA), but not salicylic acid (SA), is involved in the upregulation of thiamine biosynthesis in the plant response to abiotic stress.

## Results

### Increased expression of thiamine biosynthesis genes during early response of Arabidopsis seedlings to oxidative, salt and osmotic stress

**Figure 2:**
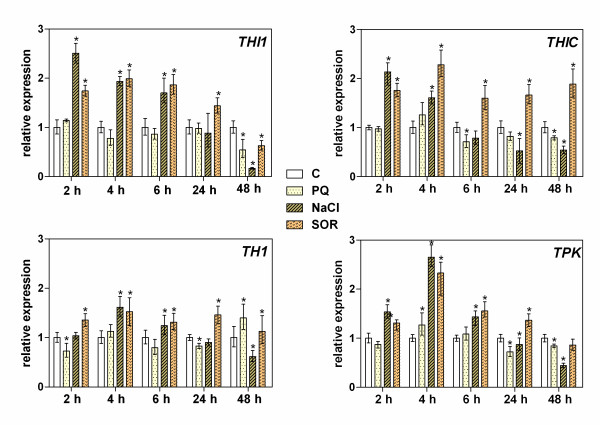
**Time-dependent response of Arabidopsis seedlings to oxidative, salt and osmotic stress revealed through thiamine biosynthesis gene expression changes**. Nine day old seedlings of *A. thaliana *ecotype Columbia were transferred onto MS medium supplemented with 0.2 μM paraquat (PQ), 200 mM NaCl or 200 mM sorbitol (SOR) for the indicated times. Transcripts of the *THI1 *(coding for HET-P synthase), *THIC *(coding for HMP-P synthase), *TH1 *(encoding thiamine monophosphate synthase/HMP-P kinase bifunctional enzyme) and *TPK *(coding for thiamine pyrophosphokinase) genes were quantified in seedling leaves by RT-PCR and expressed relative to the corresponding gene expression levels in the control seedlings grown under stress-free conditions. The asterisk indicates a statistically significant difference when compared with the control samples (*t*-test, *P *< 0.05).

The expression of the four main genes involved in thiamine biosynthesis in *A. thaliana *was analyzed during different phases of the responses (early sensing and later adaptation) to oxidative (0.25 μM paraquat; PQ), salt (200 mM NaCl) and osmotic (200 mM sorbitol; SOR) stress conditions. These tests assessed the genes responsible for the biosynthesis of the pyrimidine and thiazole moieties of thiamine (*THIC *and *THI1*, respectively), the coupling of HET-P and HMP-PP to form TMP (*TH1)*, and the activation of thiamine to TDP (*TPK*). A fast sensing response (within 2 h) by Arabidopsis seedlings to environmental changes (Figure 2) was observed through increases in the expression of two genes involved in early steps of thiamine biosynthesis, *THI1 *and *THIC *(2.9- and 2.2-fold increases, respectively) in the presence of high concentrations of salt or SOR, but not PQ. In the case of a longer stress duration of up to 48 h, the higher expression of *THIC *in the presence of SOR was similar (2-fold higher expression as compared with unstressed plants) but was found to be dramatically decreased under persistent high salt conditions. The stress-induced changes in the expression of *THI1 *showed a similar time-dependence, with the notable exception of osmotic stress for 48 h during which time the expression of this gene decreased in contrast to the response of the *THIC *gene. The expression of *TH1 *was also altered by these stress conditions with a time profile similar to that of THIC but with increases of no more than 1.5-fold. The highest differential expression response (1.5- to 3.0-fold) of the *TPK *gene was observed after 2-4 h from the application of the stress stimulus (SOR or NaCl) to the growth medium.

**Figure 3:**
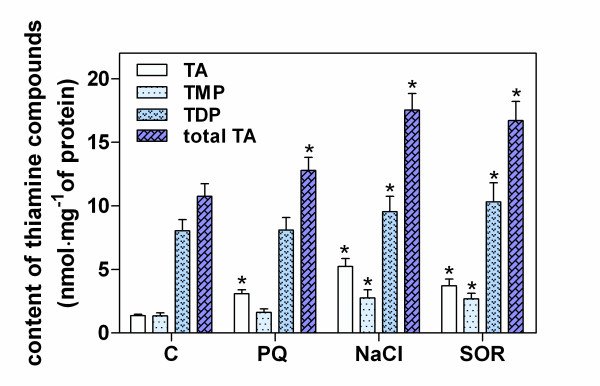
**Changes in the levels of thiamine and its phosphate esters in Arabidopsis seedlings in response to abiotic stress conditions**. Nine day old seedlings were maintained for 48 h on growth medium supplemented with 0.2 μM PQ, 200 mM NaCl or 200 mM SOR. Thiamine compounds were assayed in seedling leaves by RP-HPLC via post-column oxidation to thiochrome derivatives and fluorometric detection. The asterisk indicates a statistically significant difference when compared with the control samples (*t*-test, *P *< 0.05).

Supplemental experiments were performed that investigated changes in the overall thiamine contents in the leaves of Arabidopsis seedlings grown under stress conditions for 48 h (Figure 3). The total thiamine levels, i.e. the sum of the thiamine, TMP and TDP contents, were found to increase in the seedlings under stress conditions by 160-170%. Whilst TDP predominated over the other thiamine compounds under all of the tested conditions, its level increased by only 120-130% during the plant adaptation stress response. Hence, the observed increase in the total thiamine level is principally due to the large increases in TMP and free thiamine (by 200-380%, as compared with unstressed plants).

### Early response of TDP-dependent enzymes to oxidative, salt and osmotic stress

**Figure 4:**
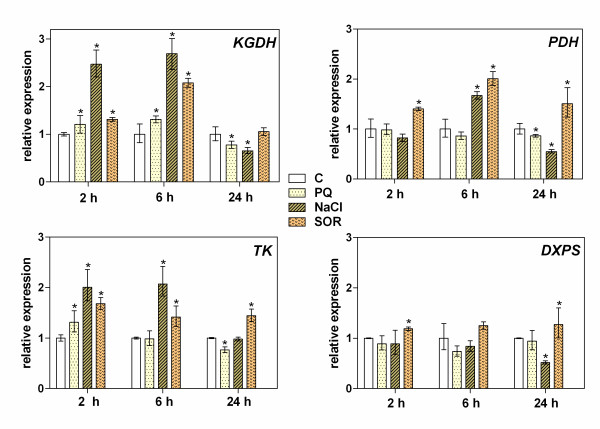
**Time-dependent changes in the expression of TDP-dependent enzyme genes: E1 components of α-ketoglutarate dehydrogenase (KGDH) and pyruvate dehydrogenase (PDH) complexes, transketolase (TK) and 1-deoxy-D-xylulose-5-phosphate synthase (DXPS)**. Gene expression was determined by RT-PCR analysis of the leaves of nine day-old seedlings maintained for the specified time on medium supplemented with PQ, NaCl or SOR or without any stressor (C). The asterisk indicates a statistically significant difference when compared with the control (C) samples (*t*-test, *P *< 0.05).

In an attempt to correlate the stress-induced increases in the expression of plant genes involved in thiamine biosynthesis with the actual requirement of the plant for TDP under different stress conditions, we analyzed the expression level of several genes encoding TDP-dependent enzymes that play critical roles in cellular metabolism (KGDH, PDH, TK and DXPS). In these experiments, KGDH appeared to be most sensitive to environmental changes. In the case of this enzyme, a 2 to 3-fold increase in expression was observed by 2 h (salt) or 6 h (SOR) after exposure to stress conditions (Figure 4). Over a longer time scale (24 h), this expression returned rapidly to a basal level. The upregulation of TK genes (about 2-fold) was also observed with a similar time-dependence under salt stress, but in the presence of SOR this expression increase was prolonged for 24 h. PDH genes were also found to be involved in salt and SOR stress sensing. However, the expression increases in these genes (about 2-fold) were highest after 6 h under both stress conditions. In contrast, only a marginal (1.2-fold) increase in DXPS gene expression was observed after 6 h of salt or SOR treatment, and these levels then remained constant for 24 h.

### Involvement of plant hormones in the up-regulation of thiamine biosynthesis during early response to stress

**Figure 5:**
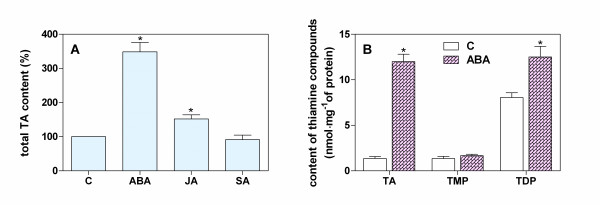
**Changes in the levels of total thiamine (**A**) and of individual thiamine compounds (**B**) in the leaves of Arabidopsis seedlings treated for 48 h with various plant hormones**. Seedlings were treated with 100 μM abscisic acid (ABA), 100 μM jasmonic acid (JA) or 200 μM salicylic acid (SA) and assayed for thiamine compounds as described in Figure 3. Seedlings maintained on growth medium without any added hormone served as the control (C). The asterisk indicates a statistically significant difference when compared with the control samples (*t*-test, *P *< 0.05).

**Figure 6:**
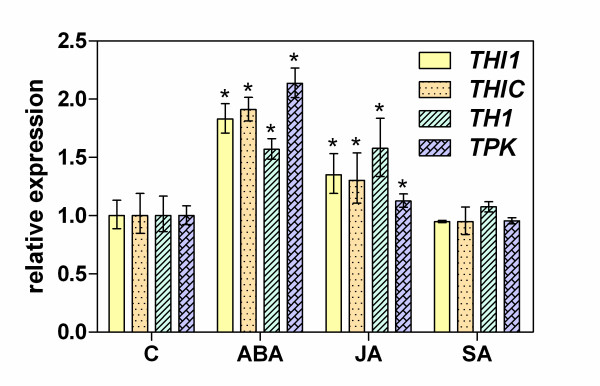
**Effects of plant hormones on the expression of thiamine biosynthesis genes in Arabidopsis seedlings**. Nine day old seedlings were kept for 6 h on growth medium supplemented with 100 μM ABA, 100 μM JA or 200 μM SA. Gene expression in seedling leaves was analyzed by RT-PCR. The asterisk indicates a statistically significant difference when compared with the control samples (*t*-test, *P *< 0.05).

The results of control analyses of thiamine pools in Arabidopsis seedlings grown for 48 h on standard medium supplemented with plant growth regulators, including ABA, jasmonic acid (JA) and SA, are presented in Figure 5A. The total thiamine content was markedly increased in the presence of ABA (360% of the control levels), but was much less enhanced (140%) by JA and did not exceed control levels in the presence of SA. TMP, produced *de novo *under the influence of ABA, was found to accumulate mostly in a free thiamine pool (8.8-fold increase over the control), probably owing to the activation of unspecific phosphatases. However, the TDP level was essentially maintained as only a 1.5-fold increase was observed (Figure 5B). These results were further confirmed by determinations of the thiamine biosynthetic gene transcript levels after 6 h following ABA supplementation (Figure 6). Remarkably, the expression of all analyzed genes increased in seedlings grown in ABA-containing medium: 2-to 2.2-fold increases were noted for the *THI1*, *THIC *and *TPK *genes and a slightly lower (1.5-fold) upregulation was observed for the *TH1 *gene. In contrast, over the same time scale, the presence of SA in the medium did not affect the thiamine biosynthesis processes and only a slightly increased expression (1.2- to 1.5-fold) of *THI1*, *THIC *and *TH1*, but not *TPK*, was observed after supplementation of the medium with JA.

**Figure 7:**
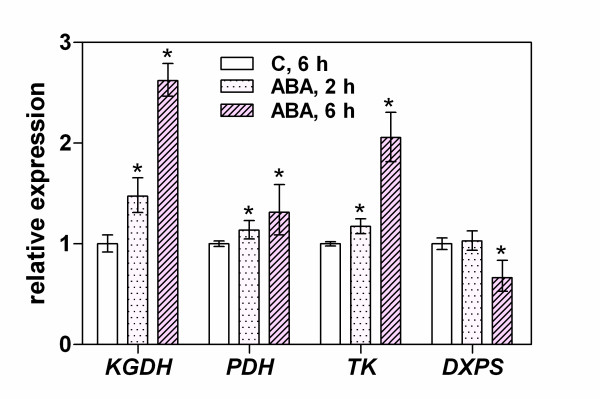
**Effects of ABA on the expression of genes coding for TDP-dependent enzymes in Arabidopsis seedlings**. Nine day old seedlings were maintained for the specified times on medium supplemented with 100 μM ABA. Gene expression in seedling leaves was analyzed by RT-PCR. The asterisk indicates a statistically significant difference when compared with the control samples (t-test, *P *< 0.05).

An analysis of the effects of medium supplementation with ABA on the transcription of TDP-dependent enzymes (Figure 7) revealed KGDH and TK (2.6- and 2.0-fold expression increases, respectively) as the enzymes likely to be regulated by ABA. However, we did not observe any significant influence of ABA on PDH expression in our present experiments.

**Figure 8:**
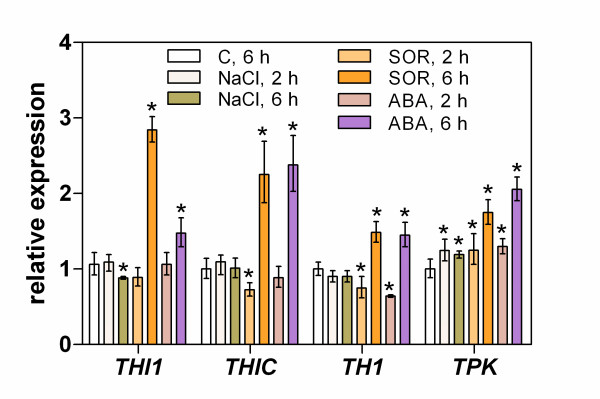
**Changes in the expression of thiamine biosynthesis genes in the seedlings of an ABA-defective Arabidopsis mutant subjected to salt or osmotic stress or treated with exogenous ABA**. Nine day old seedlings of the *aba1 *mutant of *A. thaliana *(ecotype Landsberg erecta) or of the corresponding wild-type plant (C) were maintained for the indicated time on medium supplemented with NaCl, SOR or ABA. Gene expression in the seedling leaves was analyzed by RT-PCR. The asterisk indicates a statistically significant difference when compared with the control samples (t-test, *P *< 0.05).

To further confirm the involvement of ABA in the upregulation of thiamine biosynthesis during water stress sensing, a mutant defective in ABA biosynthesis (*aba1*) was analyzed. The expression of thiamine biosynthesis genes in *aba1 *mutant plants treated with SOR showed a similar pattern to that in the wild type plants (Figure 8), with the only noted difference being that the response was slower in the mutant plants (6 h versus 2 h in the wild plants). In contrast, under salt treatment, the *THI1 *and *THIC *genes were not significantly affected in this mutant plant and *TH1 *and *TPK *expression showed a wild-type pattern.

## Discussion

Because their growth and development are affected by environmental fluctuations, plants require fast responses to these changes for the maintenance of internal homeostasis. Hence, a number of sensitive mechanisms have evolved in plants to allow rapid sensing of environmental cues and facilitate growth modifications for adaptation and survival [21,22]. As stress has an impact on the carbon fixation and overall energy status of plants, most previous genetic, proteomic and metabolic studies of this area have dealt with changes in glycolysis, the tricarboxylic acid cycle, the pentose phosphate pathway and photosynthesis [23-25]. However, these earlier reports have presented data that was obtained under very different growth or stress conditions, and also over quite different time scales and using different measurement parameter. This makes it difficult to compare the results from different laboratories in a meaningful way.

In our current study, which for the first time evaluates the early response stage ("stress sensing") rather than the long-term adaptation to stress in plants, we compared (i) the expression levels of the genes involved in thiamine/TDP biosynthesis pathways; (ii) the expression levels of genes coding for the most important TDP-requiring enzymes; and (iii) the thiamine and its phosphate ester contents, in Arabidopsis seedlings treated with abiotic stressors including PQ, NaCl and SOR. The application of NaCl or SOR into the plant growth media during the first few hours of treatment is sensed by the plants as general water stress rather than as salt- or SOR-specific effects [26]. It is generally accepted, that stress-generated damage involves the most important pathways and hence an obvious need to upregulate the main biosynthetic processes, including the biosynthesis of TDP. Indeed, we observed in our present experiments that the expression of all TDP-biosynthesis genes had already been increased at 2 h after the application of two water-stress agents (Figure 2). Interestingly, these genes showed decreased expression under salt stress over a longer time scale of several days, suggesting an extensive ion penetration into plant tissues [26] that could possibly generate new stimuli and adaptation processes. However, in our SOR stress experiments, the most of the early observed changes, particularly those involving the *THIC *gene, were maintained over a long time scale. This may indicate that a hormonal signaling mechanism rather than water availability controls plant growth under these conditions [27]. Surprisingly, we did not observe any marked influence of PQ on the expression of TDP biosynthesis genes over a time scale of a few hours, which is in contrast to the findings of an earlier study of long-lasting PQ-generated oxidative stress [20].

Our observed increases in gene expression during initial stages of the plant stress responses may indicate that (i) the translated proteins are susceptible to damage under unfavorable conditions and an increase of transcription is therefore required to maintain the proper thiamine cellular levels; (ii) the TDP-dependent enzymes are destroyed and, parallel to their re-synthesis, a new requirement for their cofactors emerges; and/or (iii) thiamine production is necessary to overcome the consequences of stress or to develop new adaptation strategies. Our analysis of thiamine and its phosphate analogs in stressed plants (Figure 3) showed that the observed changes in the expression of thiamine biosynthetic genes correlated with the activity of the encoded enzymes. The increase in the thiamine and TDP contents during the early Arabidopsis response (sensing) to water stress is consistent with similar changes recently reported in *Z*. *mays *seedlings subjected to long-lasting osmotic, oxidative and salinity stress [19]. This suggests that during stress sensing and adaptation in plants, the biosynthesis of thiamine is tightly regulated. Moreover, this regulation does not seem to be specific to the type of stress and could benefit from two different mechanisms previously identified in plants. The first type of possible regulation occurs at the genetic level and can apply to the *THIC *gene promoter as it contains a TDP-dependent riboswitch [15]. The other regulation process can operate at the protein level, as was demonstrated in a study of *Z*. *mays*, where TMP synthase activity was strongly inhibited by an increased supply of HMP-PP, produced through the activity of the kinase domain of the same protein, THI3 [11].

To test the hypothesis that the effects of stress conditions on thiamine compound pools in plant tissues arise from the increased requirement for TDP from the TDP-dependent main catabolic pathways, we analyzed the stress-dependent changes in the expression of several genes encoding TDP-dependent enzymes (Figure 4). The mitochondrial production of acetyl CoA and the tricarboxylic acid cycle are assumed to be the first plant sensors of oxidative stress [23,28,29]. Our current analyses identified KGDH and PDH as the main source of stress perturbation. However, the upregulation of the expression of these two mitochondrial TDP-dependent enzymes can result not only from the increased requirement for mitochondrial potential restoration, respiration control, nitrogen metabolism or glutamate signaling [30] but also reflect the activation of a stress signaling response to a metabolic imbalance as a result of a KGDH side reaction resulting in reactive oxygen species production [29].

The TK enzyme, which operates in the chloroplastic Calvin-Benson cycle (CBC), is involved in carbon fixation [31] and its activity is a limiting factor for sucrose production and photosynthesis [32]. As it functions also in the pentose phosphate pathway, TK is additionally important for the generation of NADPH, cooperating with a variety of oxidant-scavenging systems [23,33-35]. The early reported increase in TK transcripts in Arabidopsis agrees with the changes in TK activity observed in *Z*. *mays *under salt and oxidative stress [19] and supports the hypothesis that this enzyme is involved in plant stress protection. DXPS is another TDP-dependent enzyme which is involved in the production of powerful antioxidants, the carotenoids [36,37]. The activation of carotenoid synthetic pathways has been observed during osmotic, salt and drought stress [38-40]. In our present study, however, no significant changes in the expression of DXPS-encoding gene were observed over 24 h of stress treatment, possibly because its regulation occurs at the posttranscriptional level [41].

The well characterized and essential roles of the phytohormones in a wide range of stress adaptive processes in plants [42,43] prompted us to search for candidate regulators of the activation of thiamine biosynthesis and TDP-dependent metabolic processes during salt and osmotic stress sensing and in the later adaptation phase. Our focus on SA was based on previous reports of an activation of a cabbage *THIJ*-like gene product involved in HMP phosphorylation via the SA-dependent signaling pathway [44] and an involvement of SA in thiamine-induced plant resistance to pathogen attacks [45]. However, our treatments of Arabidopsis plants with exogenous SA did not cause any observable changes in expression of genes involved in thiamine biosynthesis (Figures 5 and 6).

JA is associated with the defense against necrotrophic pathogens and herbivorous insects [46] but its involvement in salt stress-dependent gene regulation has also been suggested [47]. In our current study, supplementation of growth medium with JA caused a slightly increased thiamine content in Arabidopsis seedlings, resulting from the activation of thiamine biosynthetic genes, mostly *TH1 *(Figures 5 and 6). Although the mechanism underlying thiamine biosynthesis regulation by JA requires further analysis, it is noteworthy that a cis-acting element T/GBOXATPIN2 (AACGTG) was identified in the *TH1 *promoter region [48] which is responsible for activating this gene in response to JA upon wounding [49].

ABA regulates nearly 10% of the protein-coding genes in plants [50] and commonly participates in the transcriptional regulation processes that operate under salt and osmotic stress conditions [51-54]. The upregulation of the *THI1*, *THIC*, *TH1 *and *TPK *genes and the 3.5-fold increase in the total thiamine levels in Arabidopsis seedlings under the influence of an external ABA supply that we observed in this study provide the first evidence for the involvement of ABA in thiamine biosynthesis under stress conditions (Figures 5, 6). These findings are also interpretable in the light of previous analyses of *THI1*and *THIC *promoters that showed the presence of an ABA-responsive element (ABRE) in the *THI1 *promoter [16] and putative stress-related elements in the *THIC *promoter, including ABRE and drought-response element [20]. *In silico *analysis of the *TPK *promoter has identified a dehydration and ABA-inducible expression element MYBATRD22 (CTAACCA) and of the *TH1 *promoter an ABRE - ABRELATERD1 (ACGTG) element.

Our current analysis of the expression of TDP-dependent enzymes in seedlings grown on ABA-containing medium showed a transcript accumulation for KGDH- and TK-encoding genes but no significant effects on PDH gene transcripts. These data are consistent with our previous observations of the increased ABA content and TK activity in *Z*. *mays *seedlings treated with NaCl and polyethylene glycol [19]. Taken together, these data are consistent with the well established hypothesis regarding the linkages between ABA and plant mitochondrial functionality [55-57].

Although the application of exogenous ABA has often been used to mimic water stress responses in plants [58], it is generally accepted that both ABA-dependent and ABA-independent processes are involved in these types of responses [59]. In an attempt to further validate the hypothesis that ABA is responsible for thiamine biosynthesis in the early response (sensing) of plants to water stress generated by salt or SOR, a mutant Arabidopsis plant (*aba1*) was employed in our present experiments. This mutant is defective in the production of zeaxanthin epoxidase, an enzyme that catalyzes two early steps in the generation of the epoxycarotenoid precursor as part of the ABA biosynthetic pathway [60]. In the analysis, *aba1 *showed different behaviors in response to salt and osmotic stress. Under salt stress, a decrease in the *THI1 *and *THIC *expression levels in the *aba1 *mutant plants was observed, confirming the regulation of these thiamine biosynthetic steps by ABA. The expression of *TH1 *and *TPK *was found to be maintained as in the wild type plant. In contrast, in the presence of SOR the activation of thiamine biosynthetic genes was found not to be markedly different from wild type plants, suggesting that some additional, e.g. post-transcriptional, regulatory processes for thiamine biosynthesis operate under these conditions, although the details remain to be clarified.

Our present findings suggest a contribution of ABA to the transcriptional regulation of the two first genes that function in thiamine biosynthesis (*THI1 *and *THIC*) in Arabidopsis seedlings subjected to salt and osmotic stress, particularly at the early stages of the stress response (i.e. stress sensing). However, other as yet unidentified endogenous mediators must also be involved and the relative importance of ABA becomes less significant over a longer time scale (i.e. during the plant adaptation to stress). Moreover, the *TH1 *and *TPK *genes appear to be regulated by endogenous factors other than ABA, but also by ABA when it is present in the growth medium.

**Figure 9:**
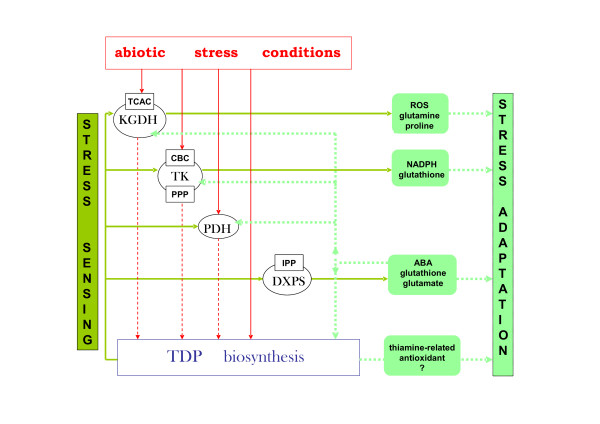
**A proposed role of TDP biosynthesis and TDP-dependent enzymes in stress sensing and adaptation in plants**. Environmental stress damages the main TDP-dependent enzymes in plants (TK, PDH, KGDH, DXPS) causing them to function as sensors of these stimuli. The further propagation of stress sensing processes activates the expression of thiamine/TDP biosynthetic enzymes (THI1, THIC, TH1 and TPK) that are necessary for the regeneration of the TDP-dependent pathways such as the Calvin-Benson cycle (CBC), the pentose phosphate pathway (PPP), the tricarboxylic acid cycle (TCAC), and the isoprenoid phosphate biosynthesis pathway (IPP). This in turn facilitates the activation of certain defense mechanisms and the production of stress protecting molecules including thiamine which can function as an antioxidant or as a stress alarmone.

On the basis of the data obtained in this study as well as the results of previous reports, a role for thiamine biosynthesis and TDP-dependent metabolic pathways in abiotic stress sensing and adaptation can be hypothesized (Figure 9). Briefly, abiotic stressors which appear in the plant environment begin to inactivate the major metabolic pathways, the crucial enzymes of which can act as stress sensing centers (i.e. KGDH, TK). The urgent requirement for the rapid reactivation of these factors generates the secondary processes or stimuli that initiate a further adaptation phase. The activation of TDP biosynthesis processes leads to thiamine/TDP overproduction, which supports defense responses such as NADPH production or carotenoid biosynthesis. TDP production and the subsequent activities of TDP-dependent enzymes come under ABA control but is not the sole regulator of these processes. However, thiamine compounds themselves can also act as a significant source of antioxidants [61,62] or play a stress alarmone function as has been suggested in bacteria under carbon starvation conditions [63].

## Conclusions

We here demonstrate for the first time that TDP biosynthesis processes are quickly activated during the early phase (sensing) of the Arabidopsis response to salt and osmotic stress. The produced TDP is incorporated into TDP-dependent enzymes that are up-regulated at the same time such as KGDH, PDH, TK, all of which are involved in the main metabolic pathways that respond to stress conditions in plants. ABA seems to be involved in the activation of genes encoding enzymes that function in thiamine and TDP biosynthesis as well as the TDP-dependent KGDH and TK genes. We propose a working model for the contribution of thiamine to the stress sensing and adaptation processes in plants.

## Materials and methods

### Plant materials, growth conditions and stress treatments

The seeds of *Arabidopsis thaliana *ecotype Columbia (Lehle, Round Rock, TX, USA) were used for all gene expression analysis performed for the detection of thiamine biosynthesis and activation processes. The seeds of the ABA-deficient mutant *aba1 (CS21) *were obtained from the Arabidopsis Biological Resource Center (ABRC, Columbus, OH, USA). For comparative analyses using this mutant, wild-type Arabidopsis plants of the background ecotype Landsberg erecta were used.

Arabidopsis seeds (100 mg) were surface sterilized by treatment for 5 min with 0.5% Tween-20, for 5 min with 70% ethanol and for 7 min with a 15% bleach solution. Water-rinsed seeds were stratified for three days at 4°C and then grown on Murashige and Skoog (MS) agar plates supplemented with 1% sucrose but depleted of thiamine, under a 12 h light/12 h dark cycle at 22°C, with a constant light intensity of 100 μmol m^-2 ^s^-1^. Nine-day old seedlings were then transferred and grown for 2-48 h on MS media containing 0.2 μM PQ, 200 mM NaCl, 200 mM SOR, 100 μM ABA, 100 μM JA or 200 μM SA. All experiments were performed with 3-5 replicates of 20 seedlings per treatment.

### RNA isolation and quantitative real-time PCR

Total RNAs were isolated from liquid nitrogen-disrupted seedling leaves using Trizol reagent (Invitrogen, Carlsbad, CA, USA), and by treatment with RNase-free DNase I (Promega, Madison, WI, USA). Further purification was performed using a PureLink RNA Mini Kit (Invitrogen). The RNA concentrations were determined using a NanoDrop ND-1000 spectrophotometer (NanoDrop Technologies, Inc., Wilmington, DE, USA) and the integrity of the RNA was assessed on a 1% (w/v) agarose gel. cDNAs were generated from 1 μg of total RNA using Invitrogen SuperScript III Reverse Transcriptase, as recommended by the manufacturer. Quantitative RT-PCR was carried out using an Rotor-Gene 6,000 system (Corbett Life Science, USA) and SYBR Green JumpStart Taq Ready Mix, in accordance with the manufacturer's instructions (Sigma, St. Louis, MO, USA). The gene-specific primer pairs, listed in Table 1 were designed by using a Primer Express 3.0 software (Applied Biosystems, Bedford, MA, USA)). The specificity of the reactions was verified by melting curve analysis. The relative mRNA level for each gene was calculated using ΔΔC_T _values in comparison with unstressed seedlings and the EF-1 gene was used as an internal control for normalization. Quantitative RT-PCR analysis was performed using at least three replicates of each cDNA sample.

**Table 1:** The list of analyzed genes and selected PCR primers used in real-time PCR.

Gene	Locus (AGI)	Forward primer (FP)Reverse primer (RP)	T_m _FP or RP(°C)
*THI1*	AT5G54770	CTGCTGAGGATTTGATTGTG	49,7
		CGATTGTGTGTGGTGGTT	48,0

*THIC*	AT2G29630	TAGCTTACCACAAGGAGAA	46,8
		CCAATGGAAAGAGCCACATCA	52,4

*TH1*	AT1G22940	GTGATCTTCCTGACTCAT	45,8
		CCAAAGTGCAACCAGTCC	50,3

*TPK*	AT1G02880	CACGATTCACTCCTCTTCTC	51,8
		AATTCGTCGTAGATGCGATTAG	52,0

*KGDH*	AT3G55410	TGCGTCAAACCGGGCTTGTT	53,8
		AGTTGGCGGGAGTTGTGGCA	55,9

	AT5G65750	TGCTACCGTCGCTTTGGGCA	55,9
		TGAAGTGACGAGGGATGACTGCG	58,8

	AT5G55070	ACCGTTCTTGTCCAGGGTGTCCA	58,8
		AGGCGGCTAGGGTGCCATCA	57,9

*PDH*	AT5G50850	TCGGTTTCGCCCTCAAGGCA	55,9
		GTGGCTCGGTCAAGCGGACG	60,0

	AT1G59900	TGGTGTGAGGCAGGAACGGG	57,9
		AGAAGGCTCTGGCATTGGGCA	56,3

	AT3G52200	CGCCCGGACTTCCTCGCTTC	60,0
		GCACGCCAAATCGCAGAATGG	56,3

*TK*	AT3G60750	GCCGTATGAGCCGTTCCAGGT	58,3
		GAGCCTTCTCCCAGCCAGCA	57,9

	AT2G45290	ATTGGAGCCGTCACACGCCC	58,3
		TGGTGGCATCACCCGGAGAG	57,9

*DXPS*	AT4G15560	TGCAAGCCATTGGACCGTGC	55,9
		GTGCGGTTGCTGCGATGTGA	55,9

*EF-1*	AT1G07920	GAGCCCAAGTTTTTGAAGA	46,8
		CTAACAGCGAAACGTCCCA	51,1

### HPLC analysis of thiamine and its phosphate analogs

Assays of thiamine and its phosphate analogs in Arabidopsis seedlings were carried out using a previously described method [19]. Briefly, plant extracts were prepared by 12% TCA treatment of frozen, ground Arabidopsis seedlings for 5 min at 4°C. After removal of the protein pellet and TCA disposal by ethyl ether treatment, the supernatant was analyzed by reverse-phase high performance liquid chromatography (RP-HPLC) with post-column oxidation using 90 μM sodium hexacyanoferrate (III) in 0.56 M NaOH and fluorescence detection of the formed thiochrome and thiochrome phosphates [64]. For RP-HPLC separation, gradient elution (0-90%B, 16 min) was used, where solvent A contained 15 mM ammonium citrate (pH 4.2) and solvent B consisted of 0.1 M formic acid and 55 mM diethylamine. The applied HPLC equipment contained a Shimadzu (Kyoto, Japan) model LC-9A HPLC pump with Shimadzu FCV-AL proportioning valve, a Knauer (Bad Homburg, Germany) model A0263 manual injector, a Merck cartridge LiChrosphere 100RP-18 column (250 × 3.4 mm), a Shimadzu model RF-535 fluorescence monitor (excitation at 365 nm, emission at 430 nm) and Shimadzu Class-VP software (version 4) for pump control, data acquisition and analysis.

## Authors' contributions

MRK designed and coordinated the study, participated in the HPLC and molecular biology experiments and prepared the manuscript; NW carried out the molecular biology experiments; MK participated in thiamine compound HPLC analysis; AKB participated in molecular biology studies and plant propagation. All authors read and approved the final manuscript.
